# Sweeten PAMPs: Role of Sugar Complexed PAMPs in Innate Immunity and Vaccine Biology

**DOI:** 10.3389/fimmu.2013.00248

**Published:** 2013-09-02

**Authors:** Ranjeet Singh Mahla, Madhava C. Reddy, D. Vijaya Raghava Prasad, Himanshu Kumar

**Affiliations:** ^1^Laboratory of Immunology, Department of Biological Sciences, Indian Institute of Science Education and Research (IISER), Bhopal, India; ^2^Department of Biotechnology and Bioinformatics, Yogi Vemana University, Kadapa, India; ^3^Department of Microbiology, Yogi Vemana University, Kadapa, India; ^4^WPI Immunology Frontier Research Center, Osaka University, Osaka, Japan

**Keywords:** innate immunity, innate sensors, sugar associated PAMPs, disease pathogenesis, vaccinology

## Abstract

Innate sensors play a critical role in the early innate immune responses to invading pathogens through sensing of diverse biochemical signatures also known as pathogen associated molecular patterns (PAMPs). These biochemical signatures primarily consist of a major family of biomolecules such as proteins, lipids, nitrogen bases, and sugar and its complexes, which are distinct from host molecules and exclusively expressed in pathogens and essential to their survival. The family of sensors known as pattern recognition receptors (PRRs) are germ-line encoded, evolutionarily conserved molecules, and consist of Toll-like receptors (TLRs), RIG-I-like receptors (RLRs), NOD-like receptors (NLRs), C-type lectin-like receptors (CLRs), and DNA sensors. Sensing of PAMP by PRR initiates the cascade of signaling leading to the activation of transcription factors, such as NF-κB and interferon regulatory factors (IRFs), resulting in a variety of cellular responses, including the production of interferons (IFNs) and pro-inflammatory cytokines. In this review, we discuss sensing of different types of glycosylated PAMPs such as β-glucan (a polymeric sugar) or lipopolysaccharides, nucleic acid, and so on (sugar complex PAMPs) by different families of sensors, its role in pathogenesis, and its application in development of potential vaccine and vaccine adjuvants.

## Introduction

The complexity of carbohydrates exhibits enormous diversity in structure and function in the living system. Carbohydrates play crucial role in fundamental biological processes such as energy requirements and modification of basic macromolecules like proteins and lipids to make them functional macromolecules for cellular functions such as cellular communication, cell-type specificity, cell signaling, and so on. The diversification of carbohydrate structure is because of branching and complexing of monomers or polymers or through formation of glycoconjugates such as glycoproteins and glycolipids. These glycoconjugates are fundamental components of both the host and pathogens and play an important role in host–pathogen interactions and provide the molecular basis for discrimination and elicitation of appropriate immune responses in the host (1–3). Pathogen associated molecular patterns (PAMPs) are molecular signature of pathogens recognized by the host germ-line encoded pattern recognition receptors (PRRs), which are classified as toll-like receptors (TLRs), retinoic acid-inducible gene (RIG)-I-like receptors (RLRs), C-type lectin receptors (CLRs), and nucleotide oligomerization domain (NOD)-like receptors (NLRs), and DNA sensors (4–8). Most of the PAMPs such lipopolysaccharides (LPS) from gram-negative bacteria, bacterial/viral nucleic acid including single-stranded RNA (ssRNA), double-stranded RNA (dsRNA), CpG rich DNA, β-glucans from fungus, and trehalose dimycolate (TDM) from *Mycobacterium* contain sugar as integral part of structure. PAMPs, which either constituted of sugar or contain sugar as a component of the complex structure can be given a new terminology as sugar complexed PAMPs (SCPs). The SCPs group of PAMPs is a largest group of PAMPs and almost all the PAMPs are grouped into it except pure lipid and protein such as flagellin. SCPs constitute essential structural and functional moieties of the pathogen that are essential for infection and establishment of disease in the host. On other hand these SCPs also play an important role in vaccine biology. The sensing of SCPs in various compartments of cells by different PRRs activates an array of biochemical reactions, leading to the activation of transcription factors such as nuclear factor kappa B (NF-κB) and interferon (IFN) regulatory factors (IRFs) for induction of inflammatory cytokine and type-I IFNs respectively. Additionally, these innate immune responses also play a pivotal role in initiation of pathogen-specific adaptive immunity via T and B lymphocytes. In this review, we will discuss different kinds of SCPs, their recognition by PRRs, and role in disease, and how these PAMPs can be explored for therapeutic application as vaccines and/or vaccine adjuvants.

## Sensing of SCPs by PRRs

Large numbers of SCPs from different classes of pathogens (bacteria, virus, and fungi) are reported and sensing of these PAMPs by different families of PRRs is described below in detail.

### Sensing of SCPs by TLRs

Toll-like receptors sense various kinds of SCPs such as LPS, ssRNA, and hypomethylated dsDNA (CpG) in different cellular compartments of the cells. The sensing of SCPs by TLRs is described in several review and listed in Tables 1 and 2 (7, 9–11). The recently discovered TLR13 is involved in sensing of bacterial 23S rRNA (12). TLR1, TLR2, TLR4, and TLR6 are plasma membrane localized TLRs, mainly sense hydrophobic SCPs (Table 1) such as LPS, while TLR3, TLR7, TLR8, and TLR9 are endosome localized TLRs, sense hydrophilic SCPs (Table 2) such as 5′-ppp-ssRNA (9, 13). TLRs are comprised of N-terminal ligand binding extra cellular domain (ECD) with 19–25 leucine rich repeat (LRRs) motifs, single transmembrane domain, and C-terminal intracellular toll/interleukin receptor (TIR) domain (14, 15). LRRs are consists of “xLxxLxLxx” where “L” stands for leucine and “x” stands for any amino acid, and it is important for recognition of PAMPs. Binding of PAMPs to LRRs leads to receptor dimerization that induces conformational changes to TIR domain for recruitment of an adaptor and activation of the signaling cascade (13, 15). Sensing of PAMPs by TLR2 induces oligomerization with TLR1 or TLR6, while TLR3, TLR7, TLR8, and TLR9 form homodimers; however, the sensing mechanism for TLR13 is not well known. It is possible that TLR13 forms a homodimer for the activation of a downstream signaling pathway. The glycolipid LPS, which is structurally constituted of lipid A, central oligosaccharide, and antigenic *O*-polysaccharide (16) is sensed by TLR4 (17, 18) (Table 1) along with co-receptors known as myeloid differential factor 2 (MD-2) and cluster CD14 (17, 19, 20). TLR2 senses a large class of bacterial SCPs such as lipoteichoic acid (LTA) (21–23), teichoic acid (TA) (21), TDM (24), peptidoglycan (PGN) (21), glycophosphatidylinositol (GPI) anchored proteins (25), lipoarabinomannan (LAM) (26), and arabinogalactan (AG) (27, 28). Nucleic acids, the pentose sugar containing PAMPs are sensed by endosomal TLRs. The bacterial and viral DNA, which are hypomethylated compared to the host DNA, sensed by TLR9 (29–31). The viral dsRNA and ssRNA are sensed by TLR3 and TLR7, respectively (32–34). Recently, human TLR8 was shown to sense viral ssRNA-derived from human immunodeficiency virus (HIV) (35, 36).

**Table 1:** **Sensing of SCPs by plasma membrane localized PRRs**.

SCPs	Sensors	Signaling axis	Immune effector response	Reference
α-Mannans	Dectin-2	FcRγ-Syk-PLCγ-(CARD9-NF-κB/MAPK/Calcineurin-NFAT)	Inflammatory cytokines	Saijo et al. (73 )
β-Glucans	Dectin-1	Syk-PLCγ-(CARD9-NF-κB/MAPK/Calcineurin-NFAT)	Inflammatory cytokines	Taylor et al. (72 )
Glycoprotein	DC-SIGN, L-SIGN, Dectin-2	Ras/Pak/Src-Raf-NF-κB	Inflammatory cytokines	Ishikawa et al. (77 ), Ludwig et al. (80), Zhao et al. (82), Svajger et al. (83)
		FcRγ-Syk-PLCγ-(CARD9-NF-κB/MAPK/Calcineurin-NFAT)	
Glycolipid	Mincle	FcRγ-Syk-PLCγ-(CARD9-NF-κB/MAPK/Calcineurin-NFAT)	Inflammatory cytokines	Ishikawa et al. (77 )
Trehalose dimycolate (TDM)	Mincle, MCL, TLR2	FcRγ-Syk-PLCγ-(CARD9-NF-κB/MAPK/Calcineurin-NFAT)	Inflammatory cytokines	Bowdish et al. (24 ), Wells et al. (74), Miyake et al. (76)
		TRAP-MyD88-TRAFs-IKKα/β-NF-κB	
Teichoic acid (TA)	TLR2	TIRAP-MyD88-TRAFs-IKKα/β-NF-κB	Inflammatory cytokines	Schwandner et al. (21 )
Arabinogalactan	TLR2	TIRAP-MyD88-TRAFs-IKKα/β-NF-κB	Inflammatory cytokines and type-I IFNs	Strohmeier et al. (27), Underhill et al. (28)
		TIRAP-MyD88-TRAFs-TBK-IRFs	
Lipopolysaccharide (LPS)	TLR4, TLR2, DC-SIGN	MyD88/TRIF/TIRAP/TRAM-IKKα/β/IKKi-NF-κB/IRFs	Inflammatory cytokines and type-I IFNs	Hoshino et al. (18), Zhang et al. (78), Takeuchi et al. (194)
		TIRAP/MyD88-TRAFs-IKKα/β-NF-κB	
		Ras/Pak/Src-Raf-NF-κB	
Lipoarabinomannan (LAM)	TLR2/TLR1	TIRAP-MyD88-IKKi-IRFs	Inflammatory cytokines and type-I IFNs	Means et al. (26)
Peptidoglycan (PGN)	TLR2	TIRAP-MyD88-TRAFs-IKKα/β-NF-κB	Inflammatory cytokines	Schwandner et al. (21 )
GPI anchored protein	TLR1/TLR2	TIRAP-MyD88-TRAFs-IKKα/β-NF-κB	Inflammatory cytokines	Campos et al. (25 )

**Table 2:** **Sensing of SCPs by cytosolic PRRs**.

SCPs	Sensors	Signaling axis	Immune effector response	Reference
Peptidoglycan	NOD1, NOD2	RIP2-NF-κB or RIP2-CARD9-MAPK-AP1	Inflammatory cytokines	Girardin et al. (49 ), McDonald et al. (50 ), Chamaillard et al. (52 )
β-Glucans	NLRP3	ASC-Caspase-1-IL-1β	Inflammatory cytokines	Kankkunen et al. (58 )
Peptidoglycan	NLRP1	ASC-Caspase-1-IL-1β	Inflammatory cytokines	Faustin et al. (55 )
CpG-DNA	TLR9, DHX9, DHX36	MyD88-IKKi-IRFs	Type-I IFNs	Hemmi et al. (29), Peter et al. (31), Zhang et al. (126)
		IPS-1-TBK-IKKi-IRFs	
5′ppp-ssRNA	RIG-I, MDA5	IPS-1-TBK-IKKi-IRFs	Type-I IFNs	Hornung et al. (42 )
ssRNA	TLR7, TLR8	MyD88-IKKα/β-NF-κB	Inflammatory cytokines and type-I IFNs	Hemmi et al. (33), Han et al. (35), Tanji et al. (36)
		MyD88-IKKi-IRFs	
23SrRNA	TLR13	MyD88-IKKα/β-NF-κB	Inflammatory cytokines	Oldenburg et al. (12)
dsDNA	DAI, IFI16, STING, AIM2, DDX41, MRE11, cGAS	MyD88-IKKi-IRFs	Inflammatory cytokines and type-I IFNs	Rathinam et al. (59), Takaoka et al. (117), Unterholzner et al.(120), Ishikawa and Barber (121), Ablasser et al. (123), Zhang et al.(126), Kondo et al. (128)
		IPS-1-TBK/IKKi-IRFs	
		ASC-Caspase-1-IL-1β	
dsRNA	TLR3, RIG-I, MDA5	TRIF-TBK-IKKi-IRFs	Type-I IFNs	Alexopoulou et al. (32), Kato et al. (38), Kato et al. (39), Kumar et al. (89), Yoneyama et al. (195)
		IPS-1-TBK-IKKi-IRFs	

### Sensing of SCPs by RLRs

Retinoic acid-inducible gene-I-like receptors, retinoic acid-inducible gene I (RIG-I), melanoma-differentiation-associated gene 5 (MDA5), and laboratory of genetics and physiology 2 (LGP2) (37), sense RNA SCPs (Table 2). Studies on RIG-I and MDA5 knockout mice demonstrate that RIG-I and MDA5 sense viral RNA from different classes of RNA viruses (38, 39) and induce antiviral immune response; however, LGP2 functions as a regulatory molecule for RIG-I and MDA5 mediated sensing of viral RNA (40, 41). RIG-I and MDA5 are comprised of two N-terminal caspase activation and recruitment domains (CARD), middle ATPase containing DExD helicase domain (DExD helicase), and a C-terminal domain (CTD). The N-terminal CARD of RIG-I and MDA5 is necessary for recruitment of adaptor and downstream signaling. RIG-I mainly senses small size (<1 kb) RNA containing 5′ppp-ssRNA moieties (42), which could be ssRNA (43, 44) or dsRNA (45), while MDA5 mainly recognizes larger size RNA (>1 kb) from various kinds of viruses and discussed in reviews (46). MDA5 recognizes internal duplex structure of RNA, while RIG-I recognizes the terminus of RNA (47).

### Sensing of SCPs by NLRs

NOD-like receptors are cytosolic PRR consisting of 22 and 24 receptors, in humans and mice, respectively (48) and categorized into four subfamilies, NLR-A, NLR-B, NLR-C, and NLR-P, where A, B, C, and P represent acidic trans-activating domain (AD), baculoviral inhibitor of apoptosis repeats (BIR), CARD, and pyrin domain (PYD) respectively. Although these NLRs sense various kinds of PAMPs (7), only SCP-sensing NLRs are in the scope of this review. Nucleotide oligomerization domain (NOD) 1 and 2, belongs to NLR-C family, sense PGN (Table 2) from bacteria (49–51), are comprised of C-terminal LRRs, central oligomerization domain, and N-terminal CARD. NOD1 senses D-gamma-Glu-meso-DAP dipeptide (iE-DAP) (49, 52); however, NOD2 senses MurNAc-L-Ala-D-isoGln (MDP) of bacteria (49, 50). NOD1 and NOD2 receptors sense a large class of bacteria and discussed in detail in several reviews (7, 9). The PYD is containing NLRP1, NLRP3, and HIN domain containing cytosolic DNA sensor absent in melanoma 2 (AIM2) sense various SCPs from bacteria (MDP, RNA, and DNA), virus (RNA and DNA), and fungi (β-glucan and mannans) as an oligomeric complex known as inflammasome (53, 54). Bacterial, viral, and fungal PAMPs can activate different NLR inflammasome complexes and AIM2 inflammasome complex (54–59). NLRP3 inflammasome is the most studied inflammasome senses β 1, 3 D-glucan (Table 2) and regulates innate and adaptive immunity, particularly B-lymphocytes mediated antibodies (Abs) responses (58). NLRP1 inflammasome senses MDP, but the exact mechanism is not well understood (55). AIM2 (also known as PYHIN4) is a member of PYHIN (pyrin and HIN-200 family) and plays an important role in sensing of microbial DNA (Table 2) is comprised of N-terminal PYD and C-terminal HIN domain. The HIN domain sense dsDNA and PYD interacts with PYD of apoptosis-associated speck-like protein containing a CARD (ASC). The complex of DNA, AIM2, and ASC activates inactive caspase-1 and forms an inflammasome complex for production of interleukin (IL)-1 family cytokines (56, 57, 59).

### Sensing of SCPs by CLR

C-type lectin-like receptors sense SCPs from various kinds of pathogens, such as parasites (60), fungi (8, 61), bacteria, and viruses (62, 63) consist of more than 1000 members (60, 64). The number of extracellular carbohydrate recognition domain (CRD) and their cellular localization classifies CLRs to type-I transmembrane, type-II transmembrane, and soluble CLRs (65); where type-I CLRs contain multiple CRD and type-II CLRs contains single CRD (66). Furthermore, based on domain organization and sequence homology, CLRs are grouped into 17 clusters (67). Among these clusters DCs associate C-type lectin (Dectin)-1 and 2 clusters are the most widely explored (8, 68, 69). Dectin-1 senses fungal β-glucan (Table 1) (8, 70–72), which is mediated through hydrophobic entrapment of β-glucan at a groove between Trp221 and His223 residue of Dectin-1 (71). Structurally Dectin-1 is comprised of a CRD that connected to transmembrane domain through a stalk region, which contains a small intracellular tail, where immunoreceptor tyrosine-based activation motif (ITAM)-like (YxxL) and tri-acidic (DED) motifs exist. “Y” represents tyrosine, “x” can be any amino acid, and “D” and “E” are glutamic acid and aspartic acid, respectively (8). Dectin-2 senses α-mannans (Table 1); mice lacking Dectin-2, challenged with α-mannans derived from *Candida albicans* do not induce cytokines suggesting that Dectin-2 plays a pivotal role in sensing α-mannans (73). Structurally, Dectin-2 is comprised of one CTLD domain, and a short transmembrane domain that is associated with ITAM containing Fc receptor γ-chain (FcRγ) receptor. Mincle and macrophage C-type lectin (MCL; also known as Clec4d) receptor (Table 1) sense TDM (74–76). Similar to Dectin-2, Mincle also contain one CTLD, a short transmembrane domain, and an attached ITAM containing FcRγ receptor; however, while MCL is a gene duplication product of Mincle and structurally belongs to the same CLR subgroup (76). Recently, it has been reported that opportunistic skin fungal pathogen *Malassezia* is sensed by Mincle and Dectin-2, where Mincle senses glucosyl-glycolipid and mannosyl-glycolipid and Dectin-2 senses *O*-linked mannobiose-rich glycoprotein; however, Mincle and Dectin-2 deficient mice were unresponsive to these ligands suggesting that Mincle and Dectin-2 sense distinct ligands from fungus *Malassezia* and induce host immune response (77). DC-SIGN (also known as CD209) a type-II CLR senses various kinds of SCPs (Table 1) such as LPS (78), G-glycoprotein from respiratory syncytial virus (RSV), E1 and E2 glycoproteins from hepatitis C virus (HCV), and glycoprotein-140 (gp-140) from HIV (79, 80). Furthermore administration of LPS in mice promotes differentiation of monocytes to DC-SIGN receptor rich DCs (81), suggesting that DC-SIGN plays a crucial role in recognition of microbial pathogens. Liver/lymph-node specific ICAM-3 grabbing non-integrin (L-SIGN; also known as CD209L) senses E2 glycoprotein from HCV (82). Sensing of glycoproteins by DC-SIGN and L-SIGN mediates viral attachment and leads to internalization of the virus into non-lysosomal compartments in host cells (80, 82, 83). MBL, the soluble CLR, senses microbial pathogens through their sugar moieties in a non-specific manner and promotes complement fixation, for which MBL forms a multiprotein complex known as collectins (84).

## PRR Signaling

After sensing of SCPs, PRRs recruit different adaptors for effector responses, which are mainly elicited through secretion of innate cytokines such as type I IFNs and inflammatory cytokines.

### TLR signaling

**Figure 1:**
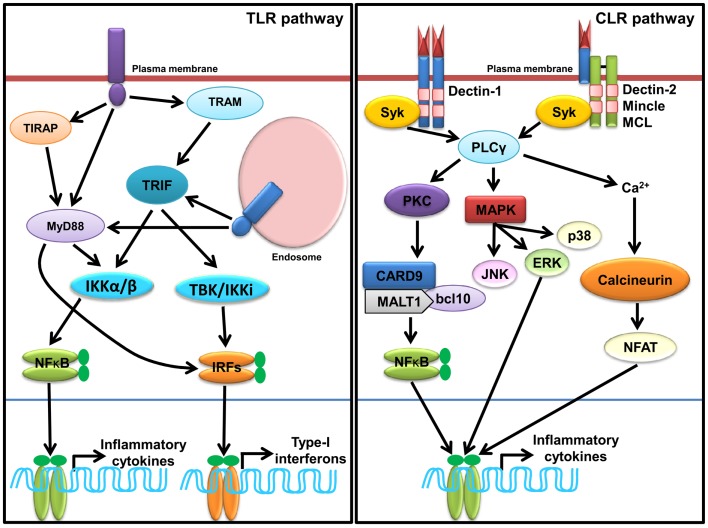
**Sensing of SCPs through membrane associated PRRs, TLRs, and CLRs**. In macrophages and dendritic cells plasma membrane localized TLRs (TLR1, TLR2, TLR4, and TLR6) and endosomal localized TLRs (TLR3, TLR7, TLR8, and TLR9) and TLR13 sense various SCPs as homo or heterodimer (shown in Table 1). TLR recruits MyD88, TRIFs, TIRAP, and TRAM adaptors for induction of inflammatory cytokines and type-I IFNs through activation of NF-κB and IRFs (IRF3 and IRF7) via IKKα/β and TBK1/IKK1, respectively. Dectin-1, Dectin-2, Mincle, and MCL are representative CLRs of Dectin-1 and Dectin-2 clusters. Sensing of various SCPs by Dectin-1 and Dectin-2 (shown in Table 1) recruits, Syk adaptor which further recruits PLCγ and activate cascade of signaling for induction of inflammatory cytokines via NF-κB and MAP kinases.

Sensing of SCPs by TLR induces conformational changes and oligomerization of receptors, which leads to the recruitment of various adaptor(s) molecules to the TIR domain, which include myeloid differentiation primary response gene 88 (MyD88), TIR domain containing adaptor-inducing IFN-β (TRIF), TIR-containing adaptor protein (TIRAP), and TRIF-related adaptor molecule (TRAM). The signaling pathways activated by different TLRs are MyD88-dependent and MyD88-independent (Figure 1) and described in several reviews in detail (7, 10, 11, 85). TLR signaling is regulated at three different levels, which include recruitment of adaptors, stability of signaling intermediates, and transcription regulation (86). A recent study shows that vitamin-D also plays a crucial role in regulation of TLR signaling (87).

### RLR signaling

**Figure 2:**
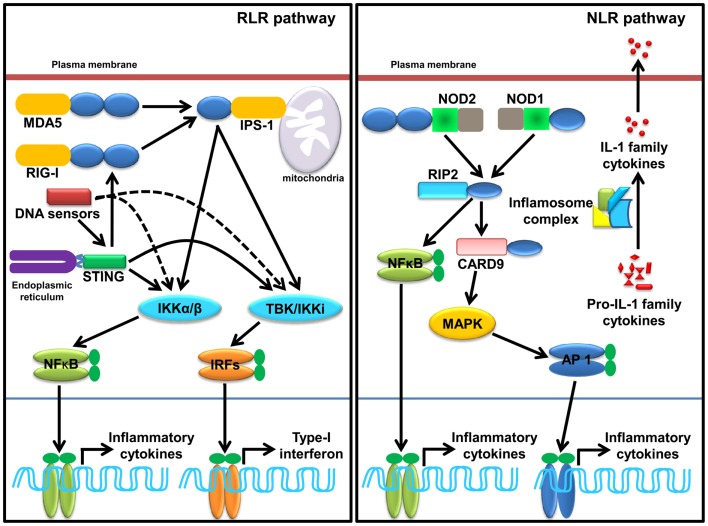
**Sensing of SCPs through cytosolic PRRs, RLRs, NLRs, and cytosolic DNA sensors**. RLRs family member, RIG-I and MDA5 sense viral dsRNA (shown in Table 2), recruits an adaptor IPS-1 for induction of type-I IFNs and inflammatory cytokines. STING, a molecule plays a pivotal role in sensing of RNA. Several DNA sensors induce inflammatory cytokine and type-I IFNs in STING dependent and independent manner. NLRs family member, NOD1, NOD2, and inflammasome (mainly NLRP1 and NLRP3 and AIM2) induces inflammatory cytokine. NOD1 and NOD2 sense various SCPs (shown in Table 2) recruit an adaptor RIP2 and CARD9 for induction of inflammatory cytokines. Activation of inflammasome complex after stimulation with appropriate SCPs (shown in Table 2) process an inactive, proIL-1 family cytokine to active IL-1 family cytokine.

Ligation of RNA to RIG-I and MDA5 promotes receptor oligomerization and recruitment of an adaptor IPS-1/MAVS/VISA/Cardiff (47, 88–90). Encounter of viral RNA with these sensors leads to the activation of sensors though accessibility of CARD for recruitment of CARD containing IPS-1 through K63 linked ubiquitination. The downstream signaling and its regulation are discussed in several reviews (37, 91–94). Recently, *in vitro* studies show that recognition of RNA by MDA5 requires CTD domains and forms head to tail oligomer strand around the RNA, leaving two CARD exposed outside, which oligomerize and recruit IPS-1, forming a string-like structure around the oligomerized MDA5 assembly (47). IPS-1 activates IKK-related kinase TBK1 and IKKi/IKKɛ, which activates transcription factor IRFs (mainly IRF1, IRF3, and IRF7), and subsequent transcription of type-I IFNs (Figure 2). It has been shown that IPS-1 is localized in mitochondria and peroxisomes and signals through IRF3 and IRF1, respectively (95). Furthermore, IPS-1 also induces production of inflammatory cytokines and requires tumor necrosis factor receptor type-1 associated death domain protein (TRADD), Fas associated protein with death domain (FADD), caspase-8, and caspase-10 (96, 97). RLR signaling is tightly regulated at multiple levels that include regulation of RNA sensing by LGP2 (40, 41), post-translational modification (PTM) of IPS-1, RIG-I, and MDA5 (98, 99), disruption of RLRs (RIG-I/MDA5)-IPS-1 signaling axis (100), destabilization of IPS-1, modulation of mitochondrial dynamics, and IPS-1 activation and regulation of downstream signaling molecules (99).

### NLR signaling

Ligand binding leads to self-oligomerization of NOD1 and NOD2 and recruits an adaptor Receptor-interacting serine/threonine-protein kinase 2 (RIP2). RIP2 is a serine threonine kinase and activates transforming growth factor activated kinase-1 (TAK-1) that subsequently activates NF-κB and mitogen-activated protein kinases (MAPKs) such as p38 and c-Jun N-terminal kinases (JNKs) (Figure 2). MAPKs is activated through an adaptor CARD9 (7, 101–104). NLR such as NLRP1 and NLRP3 induce IL-1 family cytokines, where ASC functions as a signaling adaptor and induces caspase-1-dependent maturation of IL-1 family cytokines and plays a crucial role in inflammatory responses and pyroptosis (103, 105, 106). Among all known inflammasome, NLRP3 is the most investigated inflammasome and it can be activated by multiple factors such as components of pathogenic microbes, tissue damage, autophagy, type-I IFNs, T cells, metabolic dysregulation, miRNA, and so on (107–110). Furthermore, viruses also enhance the negative regulatory mechanism of hosts to promote survival within the host (111). Activation of NLRP1 induces pyroptosis in hematopoietic cells and inhibition of NLRP1 may play a protective role in individuals having blood disorders such as anemia and leukopenia and suffering from bacterial septic shock (112). Distinct from NLRP1 and NLRP3, inflammasome AIM2 activates caspase-1 and NF-κB in an ASC dependent manner (56).

### CLR signaling

Sensing of SCPs by CLRs activates multiple signaling cascades through their own ITAMs or interacting with ITAM-motif containing adaptor proteins such as FcRγ. Signaling cascades lead to activation of NF-κB through a spleen tyrosine kinase (Syk) and CARD9 dependent pathway(s). Binding of ligands to Dectin-1 promotes phosphorylation of YxxL motifs through Syk that promotes recruitment of adaptor CARD9, which exists as a complex B cell lymphoma-10 (Bcl-10) and mucosa-associated lymphoid tissue lymphoma gene-1 (MALT-1), and subsequently activates IKK kinase complex (Figure 2). The activated IKK complex activates canonical NF-κB subunits p65 and c-Rel to induce production of multiple cytokines such as IL-6, IL-10, IL-23, IL-1β, and TNF-α through a subset of T cells such as Th1, Th2, Th17, and THF (113–115). In DCs, assembling of CARD9/Bcl-10/MALT-1 complex is dependent on paclitaxel-lipopolysaccharide complex (PLC)-γ2 receptor (8, 116). Alternatively, non-canonical activation of NF-κB takes place through Ras-RAF1 mediated phosphorylation of p65 at serine 279. Binding of activated p65 with histone acetyl transferase CBP/p300 promotes cytokines synthesis; however, its binding with b-Rel inhibits the NF-κB activity (66, 113, 115). For downstream signaling, Dectin-2 and Mincle interact with signaling adaptor FcRγ through positively charged amino acids that promote phosphorylation of ITAMs of FcRγ and subsequent recruitment of Syk. Furthermore, Dectin-2 activates NF-κB (p65–p50) and Mincle activates NF-κB in a CARD9 dependent manner (Figure 2) (115).

### SCPs and cytosolic DNA sensors

DNA-dependent activator of IFN-regulatory factors (DAI) is the first identified cytosolic DNA sensor that recognizes dsDNA and induces production of type-I IFNs (117); however, DAI-knockout cells display normal responses to poly(dA:dT), a synthetic analog of B-DNA, suggesting the existence of multiple sensors for cytosolic dsDNA (118). Nucleic acid binding protein known as leucine rich repeat Fli-I interacting protein (LRRFIPI) is a cytosolic sensor of AT rich B-DNA and GC rich Z-DNA and induces production of IFN-β, and the sensing is dependent on β-Catenin (119). Absent in melanoma 2 (AIM2), a member of the hematopoietic IFN-inducible nuclear protein HIN-200 family, has been recognized as a cytosolic DNA sensor (57) and plays a crucial role against cytosolic bacteria and DNA viruses (59). IFN-γ-inducible protein-16 (IFI16; also known as p204) induces type-I IFNs in stimulator of IFN genes (STING) dependent manner (120). STING is an endoplasmic reticulum (ER) localized dsDNA sensor and induces activation of transcription factor NF-κB and IRF3 for downstream signaling (Figure 2) (121). Sensing of dsDNA by STING is positively regulated by IFN-inducible TRIM-56, which leads to production of IFN-β (122). Recently, a nucleotidyltransferase family member known as cyclic guanosine monophosphate-adenosine monophosphate (cyclic-GMP-AMP, or cGAMP) synthase was identified as a cytosolic DNA sensor that senses DNA in a STING dependent manner. During this process cGAMP synthase (cGAS) binds to microbial DNA and synthesizes secondary messenger cGAMP that interacts with STING and activates IRF3, which subsequently leads to transcription of type-I IFNs. The crystal structure of cGAS alone and bound with DNA, ATP, and GTP demonstrates that cGAS is structurally similar to dsRNA sensor 2′–5′ oligoadenylate synthase (OAS1), but it contains a unique zinc-thumb structure through which it recognizes microbial B-DNA (123–125). DEAH (Asp-Glu-Ala-His) box containing polypeptide 9 and 36 (also known as DHX9 and DHX36) senses hypomethylated CpG DNA in Myd88 dependent manner; however, DDX41, a newly identified PRR, senses dsDNA in STING dependent manner and induces IFN-β (126). DDX41 is negatively regulated through E3 ubiquitin ligase TRIM21, where TRIM21 ubiquitinates DDX41 at K9, K48, and K115 positions, which leads to its degradation (127). Meiotic recombination 11 homolog A (MRE11) in association with DNA repair protein 50 (RAD50) recognizes various kinds of dsDNA in cytosol and induces production of type-I IFNs in a STING-IRF3 dependent manner (128). Although stimulation of granulocyte-monocyte-colony stimulating factor (GM-CSF) induces bone marrow-derived DCs (GM-DCs) with herpes simplex virus (HSV-1) and *Listeria monocytogenes* enhances type-I IFNs, siRNA mediated silencing of MRE11 does not abrogate the response, suggesting that further studies are required to understand the role of MRE11 in innate immunity (128). It can be concluded that sensing of DNA and RNA SCPs by cytosolic PRRs is more complicated than thought. Although several sensors have been discovered, the general consensus is that there may be some more cytosolic nucleic acid sensors that sense the respective DNA.

## SCPs Mediated Immune Dysregulation/Disease

Sensing of PAMPs by PRRs initiates innate immune responses through secretion of inflammatory cytokines, and subsequent B and T lymphocyte mediated humoral and cellular immune responses are essential for eradication of invading pathogens. However, excessive or hyper activation of innate and adaptive immune responses could be detrimental to the host (129, 130). SCPs are known to induce lethal immune response in the host, such as septic shock, which is characterized by a storm of inflammatory cytokines in the host after infection with gram bacteria. The LPS (also known as endotoxin) induces activation of myeloid and non-myeloid lineage of cells (131) and leads to cytokine mediated immune shock that is generally known as septic shock; the mechanism of LPS mediated immune stimulation is largely studied in laboratory animals, which is a reflective process for humans. Administration of LPS in mice induces inflammatory lesions through excessive secretion of TNF-α, IL-1β from monocytes, and macrophages that ultimately culminates in septic shock mediated death (132). LPS induces lethal effects due to TLR4-CD14-MD12 receptor complex mediated recruitment of multiple signaling adaptors (MyD88, TRAF6, TRAM, and TRIF). Septic shock is associated with progression of kidney disease with incident microalbuminuria and induces degeneration of neurons in individuals with type-I diabetes, which ultimately leads to a condition known as diabetic neuropathy (133). LPS is also shown to aggravate polyclonal B cell activation, which is a similar kind of condition that occurs in systemic lupus erythematosus (SLE). The SLE kind of effect from LPS is exerted through increased level of IgG and IgM serotype and concurrent decrease of IgA specific isotopes (134). Receptors for advanced glycation (RAGE) are involved in negative regulation of LPS induced septic shock that is mediated through reduction in LPS induced inflammatory cytokines (135). The other glycolipid constituent LTA from bacteria induces inflammatory responses that are similar to LPS and plays a critical role in bacteria pathogenesis (130).

TLR9 ligand, CpG-ODNs induces liver injury in D-Galactosamine sensitized mice that is mediated through excessive induction of TNF-α leading to mitochondria-mediated apoptosis of hepatic cells and ultimately culminates in death of the animal (129). The effect of CpG-ODNs is due to the organization of constitutive subunits, that is differential for different kind of CpG-ODNs, and some time it can be fatal to the host. Likewise, DC-SIGN receptor sensed gp120 from HIV virus inhibits secretion of inflammatory cytokine IL-6 and phosphorylation and signal transducer and activator of transcription-3 (STAT-3) that ultimately leads down regulation of host antiviral innate immune response and that further assist for viral internalization in host cell into non-endosomal compartments (136). Similarly, glycoprotein E1 and E2 from HCV modulate host cell entry factors and escape virus from neutralizing Abs. Hepatitis E virus (HEV) originated whole virion particles down regulate RIG-I mediated type-I IFNs production and employ IPS-1 in restricting antiviral inflammatory response (137). The HN series of influenza virus H1N1 can escape immune response through antigenic drift that is enabled by hemagglutinin (HA) head (138). It can be suggested that pathogens utilizes different strategies for down regulating host defense mechanism through targeting major innate immune pathways (129, 136–138). Herewith, these are the few examples to comprehend the role of SCPs in immune dysregulation and development of disease; however, proteins PAMPs are prime components involved in immune escape mechanism, which is beyond the scope of this review.

## Role of SCPs in Vaccine Biology

**Figure 3:**
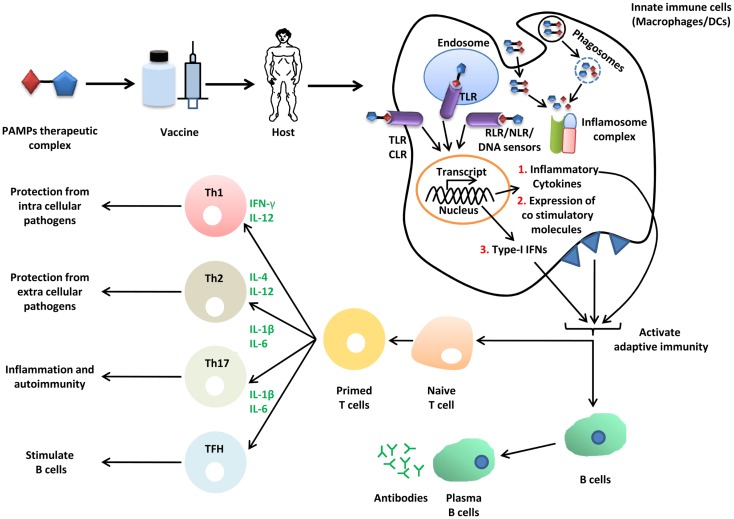
**Role of SCPs in vaccine biology**. Immunization of host (mice or human) with vaccine or vaccine adjuvant formulation PTC (PAMPs therapeutic complex) activates innate immune pathways (TLRs, CLRs, RLRs, NLRs, and DNA sensors), induce secretion of inflammatory cytokines and type-I IFNs. These cytokines further activate adaptive immune components through B and T lymphocytes. Adaptive immune memory cells protects host from infection.

The term vaccine was coined by Edward Jenner for cowpox, used for protection from small pox in humans. Formulation of vaccine can be synthetic or biological, and exerts its effect through activation of both arms of the immune system, namely, the innate and adaptive immune system (Figure 3) (139). Therapeutics agents or antigen alone do not mount appropriate immune responses for development of immunity against infection; therefore, an additional immune boosting agent known as adjuvant is required, which provides enough non-specific immune response to elicit appropriate immune response against antigenic determinants of the pathogen and protects the host from infection. Microbial PAMPs are potential candidates as vaccine adjuvants, which mainly activate innate immune cells such as macrophages and DCs. The DCs are pivotal for immunity as they link innate and adaptive immunity (140). How appropriately the immune system will be activated by a particular PAMP depends on its structural diversity and complexity. SCPs such as β-glucan (sensed by Dectin-1 and NLRP3) are made from the same basic unit of sugar but the bonds between monomeric subunits vary, which diversifies the structure. The β-glucan polymer with β 1,6 glycosidic bonds in polymer chain and β 1,3 glycosidic bonds at branching point is shown to be associated with immune stimulatory property (141). Formulation of β-glucan adjuvant with candidosis vaccine consists of α, β mannan-tetanus toxoid complex and enhances protection in mice against pathogenic *C. albicans* (142). Furthermore, β-glucan also functions as a vaccine candidate for immune clearance of fungal pathogens. Its formulation with immune adjuvant known as MF59 protects mice from vaginal candidosis infection through anti-β 1,3 D-glucan Abs (143). In addition to adjuvant and vaccine for infectious disease, β-glucan is also used for cancer therapeutics for example yeast derived β-glucan adjuvant potentially enhance antitumor effect of MUCIN-1 Abs in mice. Adjuvant and vaccine properties of β-glucan are exerted through induction of innate cytokines such as IL-6, IL-12, TGF-β, and IL-1β, and Th1 and Th2 polarization through NLRP3, MyD88, and Syk (141–143). Aforementioned studies suggest that β-glucan can be used as a potential therapeutic candidate for both infectious and non-infectious disease. Mannans (α-mannan) from fungus are Dectin-2 ligands, impart their immune response through CARD9 dependent signaling, and are involved in activation of Th1 cells (73). *C. albicans* derived mannans with α 1,6 glycosidic backbone and α 1,2 or α 1,3 glycosidic branching as well β 1,2 linked oligomer in conjugation with bovine serum albumin protects mice from fungal infection. This study suggests that similar to β-glucan, α-mannans also possess vaccine adjuvant property and hence they can be a potential vaccine candidate for protection from fungal infection (144).

For decades, LPS was known for its adjuvant property; however, the endotoxic nature renders the use of LPS in immunotherapy (145). The modified derivative of LPS known as monophosphoryl lipid A (MPLA) is less toxic and retains its immunostimulatory activity, and is used as an immunotherapeutic vaccine adjuvant (146). Co-administration of MPLA along with basic fibroblast growth factor (bFGF) enhances IgG and IFN-γ level in the serum, and show antitumor activity in mice. Similarly, formulation of MPLA with IFN-γ induces secretion of IL-12 from DCs and shows antitumor activity in mice (147). The antitumor effects of bFGF growth factor and IFN-γ are exerted through cytokine induced by DCs for activation of cytotoxic T lymphocytes (CTL). In addition, these effects are very cell-specific; for example, antitumor effects of IFN-γ are mediated through CD4^+^ T cells and selectively eliminate human leukocyte antigen (HLA) positive tumor cells (148). Furthermore, a combination of MPLA and alum is used in humans as vaccine adjuvant and it has been shown to induce CD8^+^ T cell mediated immune responses. Alum induces long-term CD8^+^ T cells; however, due to programed death (PD)-1 molecule, differentiation of CD8^+^ T memory cells to effector cytotoxic T cells is inhibited. It has been shown that in the combination of MPLA and alum, MPLA inhibits PD1 activity through IL-6 that ultimately leads to differentiation of alum primed CD8^+^ T cells into effector CTLs, suggesting its application for protection from microbial pathogens (149). A polymeric form of LPS known as SP-LPS has immuno-modulatory functions without toxicity. The conjugate of cancer drug paclitaxel and SP-LPS known as PLC shows promising antitumor effects through induction of apoptosis. Additionally, the apoptotic bodies generated during apoptosis further stimulate antigen presenting cells (APCs) and promote more efficient tumor clearance (150). Another synthetic derivative of LPS known as glucopyranosyl lipid (GLA) is shown to function as immune adjuvant. GLA, along with *Mycobacterium tuberculosis* (MTB) antigen ID83 [combination of factor of virulence (Rv2608, Rv3620) and latency Rv1813)], induces protection against MTB infection in mice. Furthermore, a GLA and MTB Ag ID3 formulation induces Th1 cytokines, suggesting that this combination can be used in humans for protection against MTB infection (151).

Mincle and TLR2 agonist TDM (also known as MTB cord factor) is shown to function as immune adjuvant. Co-administration of TDM with type III pneumococcal polysaccharide (SSS-III) in mice augments Abs response to SSS-III antigen and further protects from pneumonia infection (152). A recent genetic study demonstrated that adjuvant action of TDM activates adaptive immune responses through MCL; however, Mincle is not essential (76). The use of TDM is limited due to its toxic nature; a synthetic analog of TDM known as trehalose-6,6-dibehenate (TDB) does not show cytotoxic effects in mice. The combination of dimethyl dioctadecyl ammonium (DDA) and TDB generates a complex DDA-TDB adjuvant; immunization of mice with DDA-TDB along with MTB fusion protein (Ag85B-ESAT) shows MTB specific Th1 adaptive immune response through elevation of IFN-γ secreting CD4^+^T cells and production of isotype Abs IgG2b against the antigen (153–155), suggesting that DDA-TDB can serve as potential vaccine adjuvant against MTB. The size of DDA-TDB vesicle governs antigenic release time and overall it governs cell mediated adaptive immunity; however, the production of Abs is independent of vesicle size (156). Furthermore, TDB requires MyD88 for induction of IL-1β secretion and also activates adaptive immune response through Th17 cells (156). An *in vitro* study using mice bone marrow-derived DCs shows that TDB activates NLRP3 inflammasome in a caspase-1-dependent manner for production of IL-1β (157).

Lipoarabinomannan (sensed by TLR2 and Mincle) is shown to elicit protective immune response against *Mycobacterium* pathogenesis. Stimulation of cattle peripheral blood mononuclear cells (PBMCs) with LAM isolated from *M. avium* along with Freund’s incomplete adjuvant (FIA) induces secretion of IFN-γ. The immune stimulatory function of LAM is exerted through a MyD88 directed CARD9/NF-κB pathway and NLRP3 inflammasome activation, suggesting that *M. avium* LAM could be used as a potential vaccine candidate against bovine tuberculosis (158). PGN of bacteria is not a potent immune adjuvant; however, peptidoglycan monomer (PGM) induces appropriate immune responses without any pyrogenic and toxic effects (159). Mice sensitized with PGM adjuvant and ovalbumin antigen (OVN) formulation enhance secretion of IFN-γ and IL-4 cytokines and specific anti-OVN Abs. Similarly, PGM derived from *Staphylococcus* known as A170PG protect mice from *Staphylococcus* infection (160). The adjuvant effects of PGM are initiated through TLR2 and culminate in activation of Th1 and Th2 mediated adaptive immune responses. Most of the studies related to PGM adjuvant are performed using OVN antigen (159–161). Therefore further studies are required with a wide range of antigens from pathogens to establish the adjuvant properties of PGM.

Peptidoglycan teichoic acid (PG-TA) and LTA from *Bacillus subtilis* are non-pyrogenic at low concentration and show adjuvant activity by enhancing the number of granulocyte-monocyte-colony forming cells in bone marrow in mice. The immune stimulatory property of these SCPs is possibly mediated through GM-CSF (162), but the application of PG-TA and LTA as an immune adjuvant is limited due to poor immunostimulatory functions. The synthetic adjuvant OK-PSA prepared from the cell wall of non-virulent *Streptococcus pyogenes* strain OK-432 is structurally similar to LTA and shows potent antitumor effect in mice. Antitumor effects of OK-PSA are mediated through induction of IFN-γ, TNF-α, IL-2, IL-12, and IL-18 cytokines from Th1 cells (163), suggesting its utility as a potent immune adjuvant. Similarly, GPI anchored proteins (sensed by TLR2/TLR1) are also shown to possess adjuvant activity (164). Immunization of mice with enzymatically cleaved GPI anchored proteins along with membrane molecules from *Schistosoma mansoni* potentially protects mice from worm infection. The adjuvant effect of GPI anchored proteins induces Th1/Th2 responses via IFN-γ and TNF-α production (164), suggesting the potential application of GPI anchored proteins as an adjuvant for protection from parasite infection.

The unmethylated CpG rich DNA from bacteria and virus is a well-established deoxy pentose sugar containing SCPs. The cellular responses to CpG are mediated through its sensing by the TLR9 receptor (29). The synthetic analogs of CpG DNA known as CpG oligodeoxynucleotides (CpG-ODNs) are functionally similar to bacterial CpG DNA and show potent immune stimulatory property through induction of innate cytokines (IL-6 and type-I IFNs), improve antigen presentation by DCs, and enhance humoral and cellular immune responses (165–168). The CpG-ODNs are structurally different from bacterial DNA; backbone of bacterial DNA contains phosphodiester while CpG-ODN has a phosphorothioated (PS) backbone, which protects them from DNAse in cells. CpG-ODN contains poly G tail at 3′ and 5′ ends, due to which CpG-ODNs form high molecular weight aggregates, which is responsible for enhanced cellular uptake and potent immune responses (169, 170). Based on sequence, CpG-ODNs are classified as type A, B, and C (171, 172); however, another report shows five types of CpG-ODNs (173). Type A CpG-ODNs contain phosphodiester backbone, a central CpG dinucleotides palindrome, and a PS 3′ poly G string, activates natural killer (NK) cells, and induces high IFN-α production from pDCs but weakly induces NF-κB dependent inflammatory cytokines. Type B CpG-ODNs contain full PS backbone with one or more CpG dinucleotides and are weak inducers of cytokine IFN-α. Type C CpG-ODNs (with complete PS backbone and CpG palindrome motif) show combined property of both A and B class CpG-ODNs and efficiently stimulate B and NK cells and also induce IFN-α from pDCs. CpG-ODNs from all classes have been used as immune adjuvants. Immunization of mice with hepatitis B virus surface antigen (HBsAg) along with type B CpG-ODN (ODN 1826) enhanced HBsAg-specific IgG2a Abs (174). Similarly, CpG-ODN 1758 enhances production of tumor surface associated Id-38C13 antigen-specific IgG2a Abs in mice (175). CpG-ODN 7909 enhances hepatitis B virus (HBV) induced PBMCs proliferation (176). A study shows that CpG-ODN stimulated secretion of type-I IFNs from DCs is a dose and time dependent mechanism (177). In addition to these applications, CpG-ODNs have a wide range of application in therapeutics and discussed in several reviews (167, 178, 179).

HN series of influenza virus such as H1N1 and H5N1 lyse PBMCs in IFN-α dependent manner (180). Priming of the host immune system prior to infection can potentially protect the host from pathogenesis from influenza. Immunization of mice with β-propiolactone inactivated (this inactivation does not affect ssRNA genome of virus) H5N1 and H1N1 virus known as whole inactivated virus (WIV) protects mice from viral infection. The immune protection is mediated through TLR7 directed activation Th1 and Th2 response, as mice lacking TLR7 are susceptible to H5N1 infection (181). Furthermore, *in vitro* stimulation of human respiratory bronchial epithelial cells with GNR (gold nano rods) complexed 5′ppp-ssRNA activates RIG-I and MDA5 mediated antiviral immune memory in these cells, which further protects from infection with pandemic influenza H1N1 and common influenza A/Solomon Island/03/06 (182), suggesting the potential application of viral 5′ppp-ssRNA and WIV as vaccine candidates for protection from influenza pandemics. Poly(I:C), the synthetic analog of viral dsRNA (sensed by TLR3, RIG-I, and MDA5), shows potential antiviral immune response (183). Co-administration of poly-L-Lysine and carboxymethylcellulose along with poly(I:C) and with *Plasmodium falciparum* circumsporozoite protein (CSP) antigen increase the frequency of activation of CD8^+^ Th1 cells and anti-CSP-Abs production (184). Furthermore, immunization of mice with CpG-ODNs or poly(I:C) and alum adjuvant with HBV vaccine rAdSS1 [adenoviral vector encoding HBV S (1–223aa) and pre S (1–47aa) fusion gene] shows immune protection through CD4^+^ T cells and CD8^+^ T cells via IFN-γ and IL-12 production and inducing cytotoxic effects, suggesting their potential application in vaccine biology for protection from HBV (185, 186). Since it is known that poly(I:C) and CpG-ODNs are potent immune adjuvant, their use in combination has been shown as potential immune adjuvant functions. Mice immunized with poly(I:C) or CpG-ODN 2006 with low dose of salmonid alphavirus (SAV) antigen preparations show potent induction of type-I IFNs and further protection from SAV (187). Recently in mice and ferrets, it was shown that oral administration of an enteric capsule containing adenovirus vector expressing HA from H5N1 virus and a dsRNA adjuvant enhances antiviral immunity through anti-HA Abs; however, there is no Abs against adjuvant. Furthermore, this combination ensures the stability and slow release of viral HA antigen and dsRNA into the gastrointestinal tract, suggesting the potential application of enteric capsules as a vaccine candidate against avian influenza; however, further research work is required to understand the mechanism for immune activation (188).

Carbohydrate based targeting of CLRs (189) in APCs is an interesting strategy for therapeutic application of synthetic glucans and glycopolymer. Virus utilizes DC-SIGN for immune escape can be targeting through synthetic glyconanoparticles such as ManLAM (190–192). DC-SIGN mediated internalized glucans further activate CD4^+^ and CD8^+^ cells of adaptive immune system. Furthermore the strategy can be utilized for non-pathogenic disease like cancer (191) These synthetic glycopolymers can mimic viral glycoprotein and can be utilized efficiently as antiviral therapeutic agents (193). In summary, there are potential roads of adjuvant based vaccine development using formulations of both intact pathogen and pathogen derived SCPs or their synthetic derivatives.

## Future Perspectives

The complexity of the transcription networks that operate during SCP sensing and subsequent immune response is a major spotlight of present research. Although there is enormous progress in the field of innate immune sensing of pathogenic SCPs, there is still a lot to understand about the evolving dynamicity of protein–sugar complex (glycoproteins). Glycoproteins are the most dynamic PAMPs that pathogens utilize to escape from immune recognition and therapeutic targets. Synthetic drugs or Abs targeting protein epitopes can be masked by pathogens through protein PTM; for example, the HN series of influenza virus has evolved immune escape mechanism through the glycosylation in HA head, and during recurrence these viruses show drug resistance. Other pathogens also utilize such strategies against therapeutic targets; for example, evolution of drug resistant MTB. Therefore, it is all about the glycobiome diversity and dynamicity that pathogens explore as a survival mechanism. Though sugars are the simplest biomolecules, the bonding flexibility with the same sugar, other sugar, lipids, and proteins generates very complex structures. Understanding the sugar moieties dynamicity in pathogens is very crucial for identification of therapeutic targets. It is also necessary to understand what carbohydrate metabolic pathways are specific to pathogen survival and are not present in the host. It is also interesting to implement bio-informatics prediction method to identify putative PTM sites in structural and non-structural proteins of viruses. Pathogens target particular cells types in the host, so understanding the epigenetic changes that are modulated by pathogens in infected cells but are not occurring in uninfected cells can provide new insights in designing potential therapeutic targets. Mutation incorporated in viral genomes, genetic polymorphism of the host, and dynamicity of carbohydrate metabolism in both the host and pathogens generate a condition that decides latent or virulent stage of pathogens; for example, a third of the world population is infected with MTB, but virulence cannot be seen in all individuals. Therefore, it is very important to consider multiple factors while designing a therapeutic agent, which mainly includes comparative glycobiome analysis of the host and the pathogen and analysis of genetic makeup of the host.

## Conflict of Interest Statement

The authors declare that the research was conducted in the absence of any commercial or financial relationships that could be construed as a potential conflict of interest.

## Abbreviations

Abs, antibodies; AG, arabinogalactan; AIM2, absent in melanoma2; APCs, antigen presenting cells; ASC, apoptosis-associated speck-like protein containing a CARD; Bcl-10, B cell lymphoma-10; bFGF, basic fibroblast growth factor; BIR, baculoviral inhibitor of apoptosis repeats; CARD, caspase activation and recruitment domain; cGAMP, cyclic guanosine monophosphate-adenosine monophosphate; cGAS, cyclic-GMP-AMP synthase; CLRs, c-type lectin receptors; CRD, carbohydrate recognition domain; CSP, circumsporozoite protein; CTD, c-terminal domain; CTL, cytotoxic T lymphocytes; DAI, DNA-dependent activator of IFN-regulatory factors; DC-SIGN, DCs specific intracellular adhesion molecule (ICAM)-3 grabbing non-integrin; DDA, dimethyl dioctadecyl ammonium; Dectin, DCs associate C-type lectin; ECD, extra cellular domain; FADD, fas associated protein with death domain; FcRγ, Fc receptor γ-chain; FIA, Freund’s incomplete adjuvant; GLA, glucopyranosyl lipid; GM-CSF, granulocyte macrophage colony stimulating factor; GNR, gold nano rods; GPI, glycophosphatidylinositol; HA, hemagglutinin; HBsAg, hepatitis B virus (HBV) surface antigen; HCV, hepatitis C virus; HEV, hepatitis E virus; HIV, human immunodeficiency virus; HLA, human leukocyte antigen; HSV, herpes simplex virus; iE-DAP, D-gamma-Glu-meso-DAP dipeptide; IFI16, IFN-γ-inducible protein-16; IRAKs, interleukin-1 receptor-associated kinases; IRFs, interferon (IFN) regulatory factors; ISGs, IFN stimulatory genes; ISRE, IFN stimulatory response elements; ITAMs; immunoreceptor tyrosine-based activation motifs; JNKs, c-Jun N-terminal kinases; LAM, lipoarabinomannan; LGP2, laboratory of genetics and physiology 2; LPS, lipopolysaccharide; LRRFIPI, leucine rich repeat (LRR) Fli-I interacting protein; L-SIGN, liver/lymph-node specific ICAM-3grabbing non-integrin; LTA, lipoteichoic acid; MALT-1, mucosa-associated lymphoid tissue lymphoma gene-1; MAPKs, mitogen-activated protein kinases; MCL, macrophage C-type lectin; MDA5, melanoma-differentiation-associated gene 5; MD-2, myeloid differential factor 2; MDP, MurNAc-L-Ala-D-isoGln; MRE 11, meiotic recombination 11 homolog A; MTB, *Mycobacterium tuberculosis*; MyD88, myeloid differentiation primary response gene 88; NF-κB, nuclear factor kappa B; NLRPs, NLR family pyrin domain containing proteins; NLRs, nucleotide oligomerization domain (NOD) like receptors; ODNs, oligodeoxynucleotides; OAS1, oligoadenylate synthase 1; OVN, ovalbumin antigen; PAMPs, pathogen associated molecular patterns; PBMCs, peripheral blood mononuclear cells; pDCs, plasmacytoid dendritic cells (DCs); PD, programed death; PGN, peptidoglycan; PG-TA, peptidoglycan teichoic acid; PLC, paclitaxol lipopolysaccharide complex; PRRs, pattern recognition receptors; PS, phosphorothioated; PYD, pyrin domain; RAD50, DNA repair protein 50; RAGE, receptors for advanced glycation; RD, repressor domain; RIP2, receptor-interacting serine/threonine-protein kinase 2; RSV, respiratory syncytial virus; SAV, salmonid alpha virus; SCPs, sugar complex PAMPs; SLE, systemic lupus erythematosus; STING, stimulator of IFN genes; Syk, spleen tyrosine kinase; SIII, type III pneumococcal polysaccharide; TA, teichoic acid; TAK-1, transforming growth factor activated kinase-1; TBK1, TANK-binding kinase-1; TDB, trehalose-6, 6-dibehenate; TDM, trehalose dimycolate; TIRAP, TIR-containing adaptor protein; TIR, toll/interleukin receptor; TLRs, toll-like receptors; TNF, tumor necrosis factor; TRADD, tumor necrosis factor receptor type-1associated death domain protein; TRAF6, TNF receptor-associated factor 6; TRAM, TRIF-related adaptor molecule; TRIF, TIR domain containing adaptor-inducing IFN-β; TRIM, tripartite motif-containing protein; WIV, whole inactivated virus.
